# Investigating LGALS3BP/90 K glycoprotein in the cerebrospinal fluid of patients with neurological diseases

**DOI:** 10.1038/s41598-020-62592-w

**Published:** 2020-03-27

**Authors:** Júlia Costa, Ana Pronto-Laborinho, Susana Pinto, Marta Gromicho, Sara Bonucci, Erin Tranfield, Catarina Correia, Bruno M. Alexandre, Mamede de Carvalho

**Affiliations:** 1 Laboratory of Glycobiology, Instituto de Tecnologia Química e Biológica António Xavier, Universidade Nova de Lisboa, Avenida da República, 2780-157 Oeiras, Portugal; 2 Instituto de Fisiologia, Instituto de Medicina Molecular-Faculdade de Medicina, Universidade de Lisboa, Lisbon, Portugal; 3 Electron Microscopy Facility, Instituto Gulbenkian de Ciência, Oeiras, Portugal; 4 UniMS - Mass Spectrometry Unit, IBET - Instituto de Biologia Experimental e Tecnologica, Oeiras, Portugal; 5 UniMS - Mass Spectrometry Unit, ITQB - Instituto de Tecnologia Quimica e Biologica Antonio Xavier, Universidade Nova de Lisboa, Oeiras, Portugal; 6 Department Neurosciences and Mental Health, Hospital de Santa Maria-CHULN, Lisbon, Portugal

**Keywords:** Neuroscience, Biomarkers, Neurology

## Abstract

Galectin-3 binding protein (LGALS3BP or 90 K) is a secreted glycoprotein found in human body fluids. Deregulated levels were observed in cancer and infection and its study in neurological diseases is more recent. Here, we have investigated 90 K from human cerebrospinal fluid (CSF) of patients with amyotrophic lateral sclerosis (ALS, n = 35) and other neurological diseases (n = 23). CSF was fractionated by ultrafiltration/size-exclusion chromatography (SEC) and eluted fractions were analysed by complementary techniques including immunoblotting, electron microscopy and nano-liquid chromatography-tandem mass spectrometry. A fraction of 90 K appeared as nanoparticles of irregular shape with heterogeneous dimensions of 15–60 nm that co-eluted with extracellular vesicles in SEC. Median levels of 90 K quantified by ELISA were not different between ALS patients (215.8 ng/ml) and controls (213.3 ng/ml) in contrast with the benchmark biomarker for ALS phosphoneurofilament heavy chain (1750 and 345 pg/ml, respectively). A multiregression model supported age is the only independent predictor of 90 K level in both groups (p < 0.05). Significant correlation was found between 90 K levels and age for the ALS group (r = 0.366, p = 0.031) and for all subjects (r = 0.392, p = 0.003). In conclusion, this study unveils the presence of 90 K-containing nanoparticles in human CSF and opens novel perspectives to further investigate 90 K as potential aging marker.

## Introduction

Galectin-3-binding protein (LGALS3BP, also known as Mac-2BP or tumor-associated antigen 90 K) is a secretory protein with 567 amino acid residues^1^. It is heavily N-glycosylated with seven glycosylation sites^2^.

In early studies 90 K was purified from supernatants of tumor cells where it appeared as large molecular mass complexes^3^. Purified full-length recombinant glycoprotein from human embryonic kidney cells was found to self-associate into non-covalent oligomers of 1000–1500 kDa that appeared as ring-like structures and other shapes by electron microscopy. Recombinant 90 K also associated with collagens IV, V and VI, fibronectin and nidogen, it mediated cell adhesion and it was present in the extracellular matrix of mouse tissues^4^. 90 K was visualized by electron microscopy in nanoparticles of irregular shape found in extracellular vesicle (EV) fractions from tumor cells in culture^5^. More recently, asymmetric flow field-flow fractionation revealed novel nanoparticles from supernatants of tumor cells in culture termed exomeres, which contained 90 K^6^.

Cells from the human body as well as cells in culture release EV to the surroundings. EV include two major groups, exosomes of endosomal origin and microvesicles that derive from the plasma membrane of cells. EV can be found in body fluids, such as blood^7^ or CSF^8–11^ and constitute promising tools in disease diagnosis^12^. In view of EV heterogeneity several isolation techniques have been used including ultracentrifugation and size-exclusion chromatography (SEC)^12^.

90 K has been detected in biological fluids such as blood^1,13,14^, milk^15^, and more recent studies also reported its presence in the CSF^16,17^. In the serum 90 K was found in circulating microparticles, where it was increased for systemic lupus erythematosus and venous thromboembolism patients^18^. 90 K expression was induced in viral infection^1^ and deregulation was also found in some cancer types^13,19^. Concerning neurological diseases the information is scarce. Some evidence showed that serum 90 K was decreased in amnestic mild cognitive impairment patients compared with cognitive healthy controls^14^. On the other hand, elevated levels of 90 K were found in the CSF of fibromyalgia patients compared to controls as evaluated by nanoLC-MS/MS analysis^16^.

ALS is a neurodegenerative disease that affects motor neurons with an incidence of 2.6/100,000^20^ and where most patients die within 2–5 years after first symptoms. Most cases are sporadic (90–95%) and familial cases are less abundant (5–10%)^21^. At present neurofilaments are the most promising ALS biomarker candidates. However, they are also increased in other diseases, so the identification of other potential markers would be very useful for early diagnosis, in prognosis and in clinical trials.

In this work we have fractionated the CSF by ultrafiltration/size-exclusion chromatography and characterized the different fractions by complementary techniques for protein analysis to investigate 90 K organization in the CSF. We further quantified 90 K levels by ELISA in the CSF of patients with ALS or other neurological diseases.

## Results

### Detection of 90 K in human CSF

**Figure 1:**
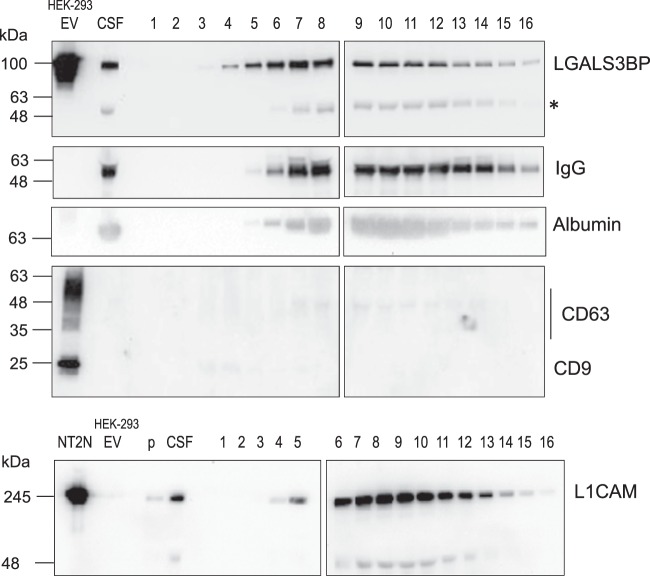
Immunoblotting of fractions from size exclusion chromatography separation of CSF. Proteins 90 K, IgG, albumin, CD9, CD63, L1CAM are shown. Results are representative of four independent purifications. CSF (5 µl 4.2-fold concentrated by ultrafiltration), fractions 1 to16 and pellet of the CSF 10,000 × g centrifugation (p) were analysed. As positive controls EV from HEK-293 cells (HEK-293 EV, 1 µg protein)^5^ and extract of NT2N cells^27^ (approximately 7.5 × 10^4^ cells) were used. Molecular mass markers are indicated on the left. * Likely corresponds to non-specific binding to IgG heavy chain. Typically 3 ml CSF were purified; for 90 K, IgG, albumin 10% of each eluted fraction were used, whereas for CD63, CD9 and L1CAM 30% were used.

The glycoprotein 90 K was detected by immunoblotting in human CSF at a molecular mass slight below around 100 kDa (Fig. 1) in agreement to serum^3^ or cell culture supernatants^5^. The mass predicted from the amino acid sequence (65 kDa) is lower than the apparent mass in SDS PAGE, which can be attributed to 90 K being heavily N-glycosylated. Since 90 K has previously been found as aggregates, in fractions of EV and in nanoparticles from serum or from cell culture supernatants^2,3,5^, we aimed at investigating its organization in human CSF. With that purpose a combination of centrifugation/ultrafiltration/SEC has been used. CSF was successively centrifuged at 500 × g and 10,000 × g to remove cells and debris and the resulting supernatant was concentrated by ultrafiltration using a cut-off of 3000 Da (typically 12-fold) to avoid loss of any proteins. Concentrated supernatant was clarified by centrifugation at 10,000 × g and applied onto qEVsingle size exclusion columns, which are used for EV purification. Sixteen fractions were collected, analysed for absorbance at 280 nm, SDS-PAGE and dynamic light scattering (Supplementary Fig. 1), by immunoblotting (Fig. 1, Supplementary Fig. 2) and by peptide mapping by nanoLC-MS/MS (Supplementary Table 1).

**Figure 2:**
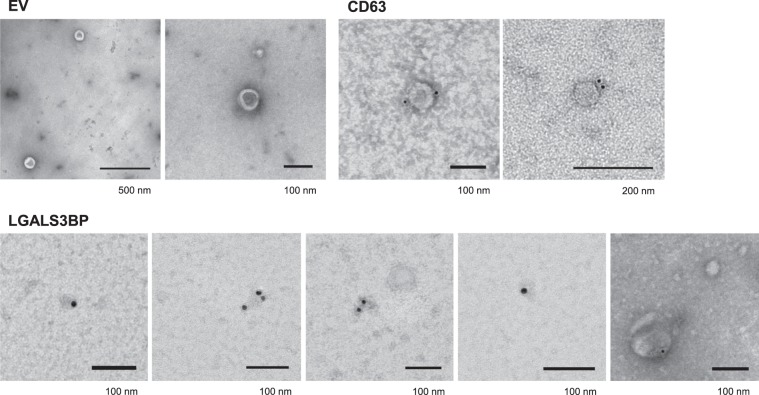
Electron microscopy analysis of EV fractions from the CSF. Fractions 3 + 4 were analysed by negative staining (EV). Immunocytochemistry with anti-CD63 and anti-90 K antibodies were performed for fractions 2–6. The dimension of the bar is indicated under the figure.

Fraction 1 consisted of the void volume, whereas large nanoparticles, such as EV, were expected to elute immediately after. Indeed commonly used EV markers CD63 and CD9 were consistently detected by immunoblotting in fractions 3 and 4 (Fig. 1). Another EV marker, annexin A2 (Vesiclepedia data base; www.microvesicles.org)^22^ was also detected only in fractions 3 and 4 by peptide mapping by nanoLC-MS/MS (Supplementary Table 1). Consistent with the presence of EV in fractions 3 and 4, DLS analysis showed the presence of nanoparticles with size around 150 nm (Supplementary Fig. 1C). In agreement electron microscopy analysis of fractions 3 + 4, showed the presence of EV with characteristic shape and size typically between 55 and 165 nm (Fig. 2) with an average diameter for the EV of 94 ± 31 nm (n = 30). In line with the immunoblotting analysis, CD63 was visualized by immunocytochemistry at the surface of EV around 80 nm from the CSF (Fig. 2). On the other hand, elution of soluble proteins, such as IgG (150 kDa) or albumin (67 kDa) was higher in later-eluting fractions (Fig. 1). Therefore, it was concluded that EV eluted immediately after the void volume whereas soluble proteins eluted in later fractions as expected from the SEC technique used.

Concerning 90 K, it started to be detected already in fraction 2 by the highly sensitive technique nanoLC-MS/MS (Supplementary Table 1) and in fraction 3 by immunoblotting and its elution peaked around fraction 7, whereas IgG or albumin peaked in later fractions (Fig. 1). To investigate its organization in early-eluting fractions, fractions 2–6 were analysed by immunocytochemistry (Fig. 2). 90 K was specifically detected in nanoparticles of irregular shapes and heterogeneous sizes typically between 15 and 60 nm; some of these nanoparticles had ring-like appearance. Occasionally 90 K also appeared on EV of variable dimensions (Fig. 2).

Other proteins of relevance in neural EV have also been analysed (Fig. 1, Supplementary Table 1). For example, L1CAM is produced by neural cells and it has been detected in the CSF^23^. L1CAM is a glycosylated cell adhesion molecule that is found at the plasma membrane and in EV where it is cleaved by metalloproteases and released to the environment^24,25^. Plasma EV of neuronal origin have been purified by affinity using anti-L1CAM monoclonal antibody (clone UJ127)^26^. Here, we tested monoclonal antibody clone UJ 127.11 to detect L1CAM from the CSF using NT2N human neuron cell extract as positive control^27^. During CSF fractionation most L1CAM eluted around fraction 8 probably corresponding to a cleaved form of the protein. The presence of L1CAM in EV from early eluting fractions was not clearly detectable by immunoblot probably due to a low amount (Fig. 1). More sensitive analysis by nanoLC-MS/MS did not show the presence of L1CAM in any fraction but the related homologue neural cell adhesion molecule L1-like protein was found in fractions 4 to 10. NCAM1, which has also been detected in EV (Vesiclepedia^22^) started to be detected in fraction 5 (Supplementary Table 1).

Transthyretin, which has previously been found associated with EV from different sources including plasma (Vesiclepedia^22^) was detected in fractions 3–16 (Supplementary Table 1). The fraction of transthyretin that elutes early may be associated with EV from the CSF.

Proteins already described in the CSF^11^ were found in the different fractions from our proteomics analysis (Supplementary Table 1). For example, beta-trace protein (prostaglandin-H2 D-isomerase) an abundant locally synthesized protein has been detected.

### Quantification of 90 K glycoprotein in human CSF

**Figure 3:**
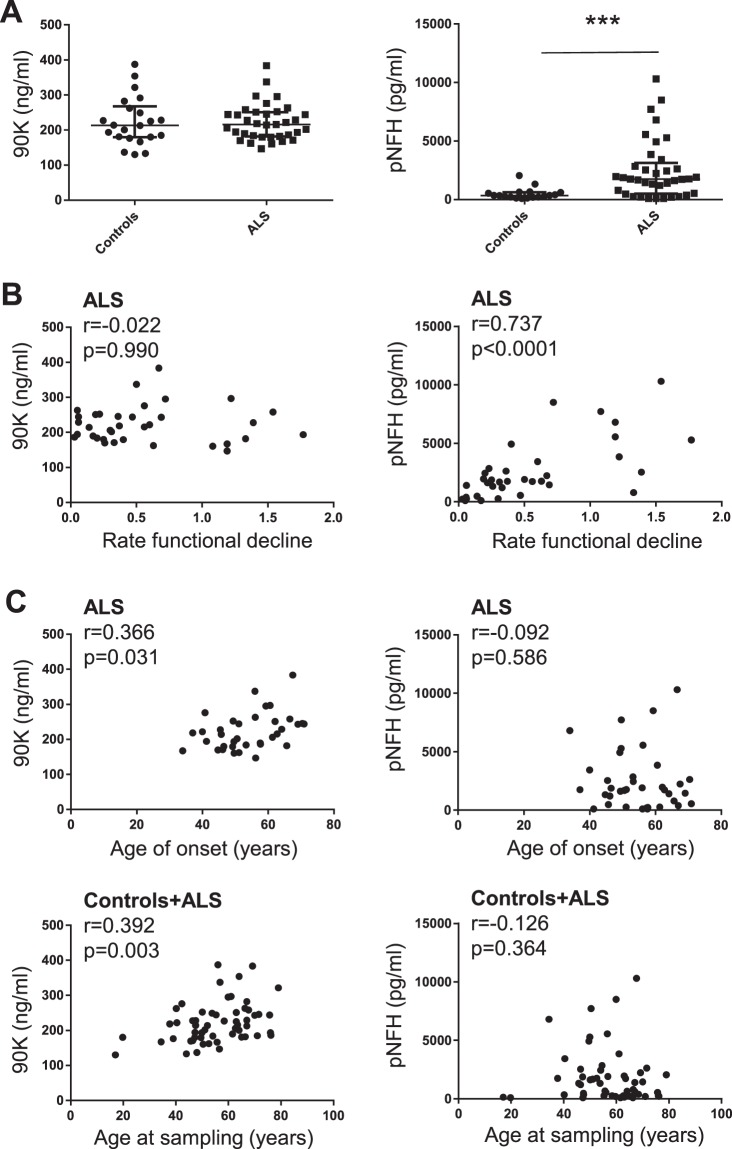
90 K and pNFH levels from the CSF of ALS patients and controls. (**A**) Comparison of 90 K and pNFH concentration in ALS and controls. (**B**) Correlation between 90 K or pNFH levels and rate of functional decline for ALS patients. Rate of functional decline was calculated by (40 − ALSFRS)/months with the disease. (**C**) Correlation between 90 K or pNFH levels and age of onset (for ALS patients) or age at sampling (ALS + control patients).

90 K concentration has been quantified by ELISA in CSF from patients with ALS (35) and a control group containing patients with several other neurological diseases (23) (Table 1). The levels of 90 K for the ALS patients (median and IQR 215.8 (181.9–251.2) ng/ml) were not different from the control group (median 213.3 (179.3–267.2) ng/ml) (Fig. 3A). This result was in contrast with pNFH, an increasingly accepted biomarker for ALS, where significantly higher levels were found for the ALS patients (median and IQR 1750 (506.0–3137) pg/ml) than for controls (median and IQR 344.6 (208.7–614.4) pg/ml (p = 0.0002). pNFH has also been found to correlate with rate of functional decline (ALSFRS scale) in this group (r = 0.737, p < 0.0001), which is in contrast with 90 K (Fig. 3B). There was no correlation between 90 K and pNFH levels for the ALS patients. Therefore, the results support the role of pNFH as marker for ALS diagnosis and disease progression but not 90 K.

**Table 1:** Demographic and clinical data of the patients included. Median and interquartile range are shown.

	Gender (M/F)	Age (years)	Disease duration (years)	ALSFRS-R	Onset site (spinal/bulbar)	pNFH (pg/ml)	90 K (ng/ml)
Controls	12/11	55.9 (43.1–65.5)	—	—	—	344.6 (208.7–614.4)	213.3 (179.3–267.2)
ALS	25/10	56.0 (47.5–66.3)	1.0 (0.6–1.8)	35.0 (32.0–37.5)	32/2	1750 (506.0–3137)	215.8 (181.9–251.2)
p	—	0.7812	—	—	—	0.0002	0.8517

In the ALS group we found a significant correlation between 90 K levels with age (r = 0.366, p = 0.031) (Fig. 3C), but in controls there was a non-significant trend (r = 0.409, p = 0.066). Merging both groups (controls+ALS) the correlation with age was significant (r = 0.392, p = 0.003) (Fig. 3C). On the other hand pNFH values did not correlate with age in any of the groups considered. A multiple linear regression model (backward method) including age and gender identified age as the single independent predictor of 90 K levels in both ALS group (b = 1.66, CI: 0.068–3.252, p = 0.041) and controls (b = 2.35, CI 0.376–4.323, p = 0.022). These results suggest that 90 K may be a potential marker of aging contrary to pNFH.

90 K or pNFH did not correlate with the functional status (ALSFRS scale). On the other hand, for an ALS group (n = 12) for which respiratory function was assessed, there was significant correlation of pNFH with respiratory function (O2 nocturnal saturation on oximetry) (r = 0.601, p = 0.043) but no correlation was detected for 90 K (r = −0.0448, p = 0.148).

## Discussion

90 K has long been identified in biofluids such as blood and it is known to be secreted by tumor cells but its relevance in the CSF started to be explored only more recently. Here, we report its detection in the CSF by immunoblotting, which validates the previous identification from other authors by nanoLC-MS/MS^16^ and by ICPL LC-MS analyses^17^. Furthermore, we calculated the levels of the protein in the CSF by ELISA around 215 ng/ml for ALS patients as well as for a group of controls. Therefore, 90 K is a relatively abundant glycoprotein from the CSF within the same order of NCAM (551.3 ± 33.3 µg/l)^23^ or plasminogen (0.25 mg/l)^28^. The value in the CSF is about 1 to 2 orders of magnitude lower than that reported for serum using the same ELISA assay (healthy controls: 5.4 mg/l; supplier datasheet) or using an MRM assay (cognitive healthy controls: 24 mg/l^14^). 90 K has been found in brain tissue, e.g., an increase has been reported in the prefrontal cortex of Alzheimer’s disease patients^29^ but the expression levels in the brain are low comparatively to other tissues as described in The Human Protein Atlas^30^. Therefore, it is likely that 90 K from the CSF derives not only from brain tissue but also from the serum upon transfer via the blood-CSF barrier.

90 K has been found in serum microparticles^18^, in nanoparticles^5^ and exomeres^6^ released by tumor cells. To investigate the structure of 90 K in the CSF we used a combined approach of ultrafiltration (3 kDa cut-off membrane) with SEC using commercial qEVsingle columns. Early eluting fractions contained characteristic EV (average 94 nm diameter) that stained positive for CD63 and CD9, and also smaller nanoparticles of heterogeneous size (15–60 nm) that were enriched in 90 K. Whether the nanoparticles are solely constituted by 90 K, or if they contain additional proteins and other molecules remains to be elucidated. Data from the literature support the capacity of 90 K to form oligomers since purified recombinant 90 K from human cells at 25–50 µg/ml concentration formed large oligomers and ring-like structures of about 30–40 nm and also other shapes as visualized by electron microscopy^4^. However, interaction with other proteins *in vivo* can also be admitted. To further characterize the 90 K-containing nanoparticles additional fractionation techniques may be used. For example, asymmetric flow field-flow fractionation^6^ or immunoprecipitation followed by “omics” strategies for molecular nanoparticle characterization are interesting possibilities. In addition to its presence in nanoparticles, occasionally we also detected 90 K on EV; since 90 K is known to bind proteins that are found in EV (e.g., collagens IV, V and VI, fibronectin) (www.microvesicles.org)^22^ an actual interaction *in vivo* could be admitted mediated by molecules from the extracellular matrix.

EV have been isolated from the CSF by several techniques including ultracentrifugation^8–10^ and combination of ultrafiltration (100 kDa cut-off)/SEC (Sepharose 4 fastflow); this latter technique showed a higher yield and improved purity comparatively to ultracentrifugation^11^. Even though most 90 K from the CSF is not EV-associated according to electron microscopy visualization here, it may co-purify with EV using the commonly used strategies for EV purification.

Results from the literature indicate that 90 K may have different functions. 90 K was initially studied from tumor cells^3^ and it is relevant in cancer development and progression^31,32^. It also plays regulatory roles in the immune system; data from the literature concerning the role of 90 K in the immune response has indicated that it may have immunosuppressive as well as immunostimulatory functions depending on the biological context, and it may induce or suppress the secretion of cytokines that play roles in inflammation^1^. It is also induced during viral infections leading to IFN and pro-inflammatory response^33^. 90 K was also suggested as potential biomarker in several diseases. For example, increased levels were found in breast cancer^13^, in non-alcoholic fatty liver disease^34^, and a specific glycoform was described as short term predictor of hepatocellular carcinoma in untreated chronic hepatitis B patients^19^. Serum circulating microparticles from systemic lupus erythematosus and venous thromboembolism patients had increases of 90 K^18^. Concerning a possible relevance in neurological diseases the evidence is scarce and more recent. For example, 90 K was increased in the CSF of fibromyalgia patients^16^, and it was decreased in the serum of amnestic mild cognitive impairment patients^14^. There was also a reduction in the CSF of obese versus non-obese women with idiopathic intracranial hypertension^17^. For ALS the results presented here, using as ALS benchmark biomarker pNFH, indicate that 90 K does not constitute a biomarker of disease diagnosis or progression.

Here we found a significant correlation between 90 K and age for the ALS patients and also for all the patients (ALS + controls). In agreement other authors also found a significant correlation between serum levels of WFA-positive 90 K and age^35^ for idiopathic pulmonary fibrosis patients, but in general the information about 90 K levels and aging is scarce. The results open a novel and interesting perspective to further investigate 90 K, its potential as aging marker and the underlying functional relevance.

## Material and methods

### Cerebrospinal fluid

CSF was collected as part of diagnostic work-up. CSF was collected by lumbar puncture into polypropylene tubes without additives and immediately stored at −80 °C. In all included subjects, serology for *Borrelia burgdoferi* and *Treponema pallidum* (CSF) and retrovirus (blood) were negative^36^. We included the following groups of subjects: 37 patients with ALS; 23 controls with other neurological diseases (21 patients, including normal pressure hydrocephalus, neuropathy, diabetic neuropathy, hereditary neuropathies, mitochondriopathy, chronic inflammatory demyelinating neuropathy, brachial plexopathy, multiple sclerosis, axonal inflammatory polyneuropathy, myelitis or ganglionopathy, and 2 subjects who underwent lumbar puncture for sudden headache with normal diagnostic work-up) (Table 1). All subjects included were regularly followed at the Neuromuscular Unit of the Department of Neurosciences (Hospital de Santa Maria, Lisbon). ALS patients progressed to probable or definite disease, according to the revised El Escorial criteria^37^. At the time of CSF sampling the patients were observed and disease severity was scored by applying ALSFRS (Amyotrophic Lateral Sclerosis Functional Rating Scale)^36,38^, the revised version was not applied as some patients were evaluated by the previous version only. As inclusion criteria we established age between 18 and 80 years and informed consent. Patients with other medical conditions, on gastrostomy, taking supplements other than vitamins, symptoms of respiratory distress or cognitive changes were excluded^36^.

The research protocol respected the Declaration of Helsinki on ethical principles for medical research involving human subjects (www.wma.net/policies-post/wma-declaration-of-helsinki-ethical-principles-for-medical-research-involving-human-subjects/). Patients were above 18 years, and all signed a written informed consent before performing the lumbar puncture, which was done as part of the diagnosis workup. The protocol for investigating biomarkers in ALS was approved by the Hospital de Santa Maria Research Ethics Committee and signed an informed consent to participate.

### pNFH and 90 K quantification

pNFH from the CSF was quantified using the ELISA kit from BioVendor Research and Diagnostic Products (RD191138300R, Czech Republic), as previously described^39^. A calibration curve between 62.5 and 4000 pg/ml was performed. CSF was centrifuged at 2000 × g 10 min, RT and the supernatant was used typically at 1:3 dilution. pNFH concentration was calculated from interpolation in a four-parameter logistic (4PL) non-linear regression curve of log (concentration) versus (absorbance 450 - absorbance 630) (Graph-Pad Prism 6). For values below the lower concentration detected (62.5 pg/ml) half of this value was considered for calculation purpose. Measurements were done in duplicate and CV was in average 10%.

90 K was quantified using the Quantikine ELISA kit (R&D systems, DGBP30B; Minneapolis) following the supplier’s instructions. A calibration curve between 0.391 and 12.5 ng/ml was performed. CSF was centrifuged at 2000 × g 10 min, RT and the supernatant was used at 1:50 dilution. 90 K was calculated from interpolation on a log (concentration) versus log (absorbance 450 - absorbance 540) curve (r^2^ above 0.994). Measurements were done in duplicate and CV was in average 2.5%.

### Statistical analysis

Statistical analysis was performed with Graph-Pad Prism 6 and IMB SPSS 24. Normality was checked by the D’Agostino and Pearson omnibus normality test and some sample distributions were not normal. 90 K concentration was presented as median and the interquartile range (IQR, 25–75% percentiles). Measurements were considered outliers when they were larger than Q3 + 1.5 × IQR or smaller than Q1–1.5 × IQR. Statistical comparison was done using the nonparametric Mann-Whitney test and Spearman non-parametric correlation analysis was used. A multiple linear regression analysis using the backward method was done to identify the independent variables for 90 K. Age, gender and the categorization of the group as ALS and controls were considered in the initial analyses for the whole population. Age and gender were then considered in the regression for each group. Values of p < 0.05 were considered statistically significant.

### CSF fractionation

CSF pools (typically 3–6 ml) of ALS patients (4 independent pools) were fractionated by centrifugation, ultrafiltration and SEC. CSF was successively centrifuged at 500 × g, 10 min, and 10,000 × g, 20 min. The resulting supernatant was concentrated by ultrafiltration (typically 12-fold) on Vivaspin 500 concentrators, cut-off 3,000 (Sartorius), and further centrifuged at 10,000 × g, 10 min to remove insoluble material arising after concentration. The concentrated supernatant was applied onto qEVsingle size exclusion column (Izon Science) and eluted with PBS as described by the manufacturer. Sixteen fractions were collected, of approximately 1 ml (fraction 1; void volume) and 200–300 µl (fractions 2–16). Protein from the collected samples was precipitated with four volumes of ethanol and further analysed by immunoblotting. For transmission electron microscopy fractions were either used directly or 4 to 8-fold concentrated on Vivaspin 500 concentrators, cut-off 3,000.

### Immunoblotting

Proteins were analyzed by SDS-PAGE using pre-cast gels from Bio-Rad and transferred to polyvinyledene fluoride membranes that were blocked for 1 h with 5% defatted dry milk (Nestle) in Tris-buffered saline (TBS) with 0.1% Tween-20 (TBST). The following antibodies were used: mouse anti-CD63 (1:500; ThermoFisher), mouse anti-CD9 (1:250; BD), goat anti-human 90 K (1:2000; R&D), goat anti-Tsg101 (1:1000; ThermoFisher), mouse anti-L1CAM UJ127.11 (1:4000; Sigma), anti-human IgG-HRP (1:10000; Sigma), rabbit anti-human serum albumin (1:1000) produced against the amino acid sequence DKLCTVATLRETYGEMAD. CD63 and CD9 were separated under non-reducing conditions. Secondary antibodies were sheep anti-mouse IgG coupled to HRP (1:4000), donkey anti-rabbit IgG coupled to HRP (GE Healthcare), rabbit anti-goat IgG coupled to HRP (1:20000) (Sigma). Washings were with TBST. Detection was performed with the Immobilon Western chemiluminescent HRP substrate (Millipore).

### Dynamic light scattering

Dynamic light scattering (DLS) measurements of SEC fractions were performed in a Zetasizer Nano ZS (Malvern Instruments). As control 0.1 g/l solution of polystyrene Uniform Microspheres diameter 0.28 µm (#PS02N, Bangs Labs, Inc.) were analysed. The settings were: sample refraction index 1.4, dispersant refraction index 1.332, viscosity 0.9133, temperature 25 °C. Three independent measurements for each fraction were made. Results presented met the instrument quality criteria.

### Electron microscopy

Fractions staining positive by immunoblotting for CD9, CD63 and 90 K were added to 100 Mesh, Copper grids (Veco Grids from EMS, E0100-Cu) that were coated with 1% (w/v) Formvar in chloroform, carbon, and glow discharged. For negative staining, the samples were stained with 2% uranyl acetate for 2 min. For immunocytochemistry, fractions were fixed with 4% paraformaldehyde in PBS for 10 min. Blocking was done with 1% BSA IgG free (Sigma, A-2058) in PBS. Grids were then incubated with primary (anti-CD9 1:25, anti-CD63 1:50, anti-90 K 1:200) and secondary (12 nm colloidal gold labeled goat anti-mouse IgG 1:20 and donkey anti-goat IgG 1:20; Jackson ImmunoResearch) antibodies in blocking buffer, followed by fixation with 1% glutaraldehyde in PBS for 5 min and stained with 2% uranyl acetate for 2 min. Grids were observed at 100 kV on a Hitachi-7650 transmission electron microscope and photographed using an AMT XR41-M Midmount camera or at 120 kv on a Tecnai G2 Spirit BioTWIN Transmission Electron Microscope from FEI and photographed using the Olympus-SIS Veleta CCD Camera, a side mount 2 k × 2 k camera.

### Mass spectrometry analysis and protein identification

Gel bands were excised, reduced in 10 mM DTT (Sigma) for 45 min at 56 °C, and alkylated in 55 mM iodoacetamide (Sigma) for 30 min in the dark. The resulting sample was digested overnight with trypsin (Promega, Madison, WI, USA) at 37 °C (1:50 protein/trypsin ratio) and cleaned up with a C18 column.

Nano-liquid chromatography-tandem mass spectrometry (nanoLC-MS/MS) analysis was performed on an ekspert™ NanoLC 425 cHiPLC® system coupled with a TripleTOF® 6600 with a NanoSpray® III source (Sciex). Peptides were separated through reversed-phase chromatography (RP-LC) in a trap-and-elute mode. Trapping was performed at 2 µl/min on a Nano cHiPLC Trap column (Sciex 200 µm × 0.5 mm, ChromXP C18-CL, 3 µm, 120 Å) with 100% A for 10 min. The separation was performed at 300 nl/min, on a Nano cHiPLC column (Sciex 75 µm × 15 cm, ChromXP C18-CL, 3 µm, 120 Å)^40^. The gradient was as follows: 0–1 min, 5% B (0.1% formic acid in acetonitrile, Fisher Chemicals, Geel, Belgium); 1–46 min, 5–35% B; 46–48 min, 35–80% B; 48–54 min, 80% B; 54–57 min, 80–5% B; 57–75 min, 5% B.

Peptides were sprayed into the MS through an uncoated fused-silica PicoTip™ emitter (360 µm O.D., 20 µm I.D., 10 ± 1.0 µm tip I.D., New Objective, Oullins, France). The source parameters were set as follows: 15 GS1, 0 GS2, 30 CUR, 2.5 keV ISVF and 100 °C IHT. An information dependent acquisition (IDA) method was set with a TOF-MS survey scan of 400–2000 m/z. The 50 most intense precursors were selected for subsequent fragmentation and the MS/MS were acquired in high sensitivity mode for 40 msec. The obtained spectra were processed and analyzed using ProteinPilot™ software, with the Paragon search engine (version 5.0, Sciex). A UniProt reviewed database (20,413 entries, accessed on 08/01/2019) containing the sequences of the proteins from Human was used. The following search parameters were set: Iodoacetamide, as Cys alkylation; Trypsin, as digestion; TripleTOF 6600, as the Instrument; gel-based ID as Special factors; Biological modifications as ID focus; Search effort, as thorough; and an FDR analysis^40^. Only the proteins with Unused Protein Score above 1.3 and 95% confidence were considered.

## Supplementary information

Supplementary information

Supplementaryinformation 2
