# Supplementary material for: Investigating LGALS3BP/90 K glycoprotein in the cerebrospinal fluid of patients with neurological diseases
Source: Sci Rep. 2020 Mar 27;10:5649. doi: 10.1038/s41598-020-62592-w (PMC7101329; doi:10.1038/s41598-020-62592-w)

Investigating LGALS3BP/90K glycoprotein in the cerebrospinal fluid of patients with neurological diseases

Authors: Júlia Costa[1,*], Ana Pronto-Laborinho[2], Susana Pinto[2], Marta Gromicho[2], Sara Bonucci[3], Erin Tranfield[3], Catarina Correia[4,5], Bruno M. Alexandre[4,5], Mamede de Carvalho[2,6]

[1]Laboratory of Glycobiology, Instituto de Tecnologia Química e Biológica António Xavier, Universidade Nova de Lisboa, Avenida da República, 2780-157 Oeiras, Portugal

[2]Instituto de Fisiologia, Instituto de Medicina Molecular-Faculdade de Medicina, Universidade de Lisboa, Portugal

[3]Electron Microscopy Facility, Instituto Gulbenkian de Ciência, Oeiras, Portugal

[4]UniMS - Mass Spectrometry Unit, IBET - Instituto de Biologia Experimental e Tecnologica, Oeiras, Portugal

[5]UniMS - Mass Spectrometry Unit, ITQB - Instituto de Tecnologia Quimica e Biologica Antonio Xavier, Universidade Nova de Lisboa, Oeiras, Portugal

[6]Department Neurosciences and Mental Health, Hospital de Santa Maria-CHULN, Lisbon, Portugal

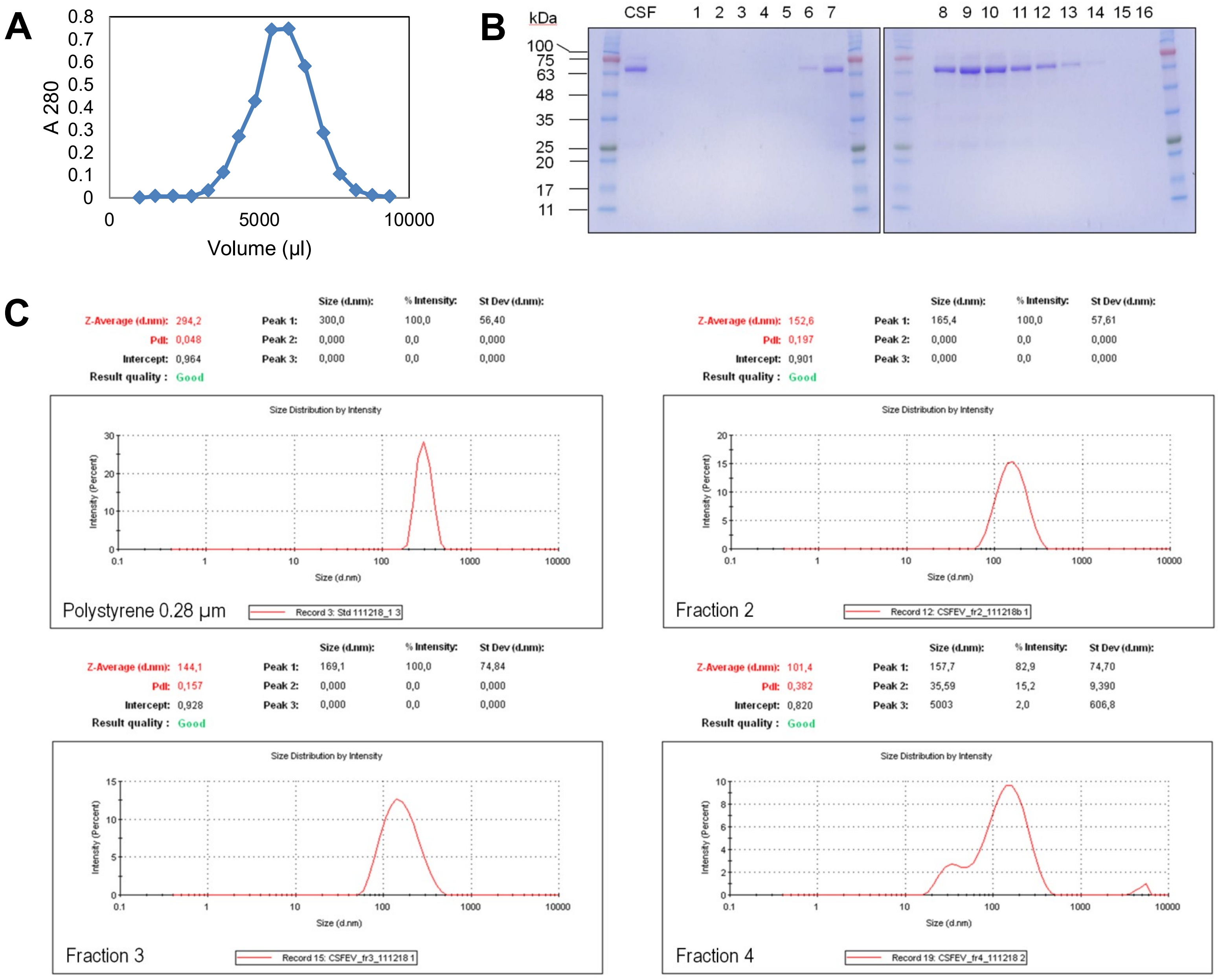

Supplementary Figure 1. CSF fractionation by size-exclusion chromatography in qEVsingle column.
A. Elution profile from the purification of an equivalent amount to a 4.5 ml CSF pool (as described in M&M). B. SDS-PAGE of eluted fractions (2% of fraction volume; 77, 87, 86, 33, 146, 200, 340, 602, 845, 374, 240, 112, 156, 102, 96 ng protein fractions 2-16, respectively) stained with Coomassie Blue R-250. C. DLS analysis of fractions 2-4.

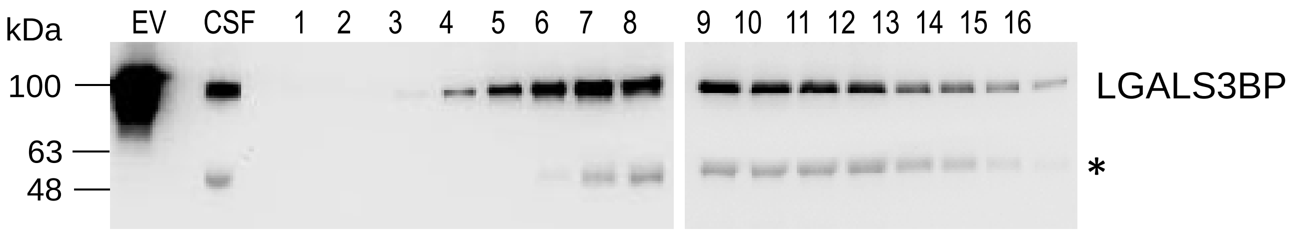

Full-length blots

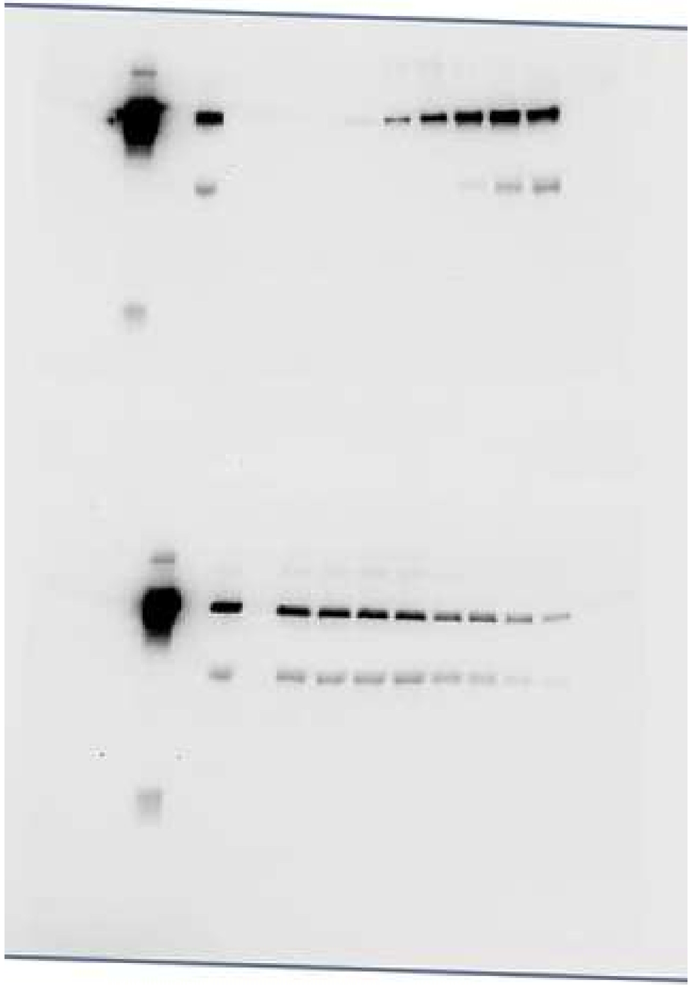

Supplementary Figure 2. Full-length blots corresponding to Figure 2.

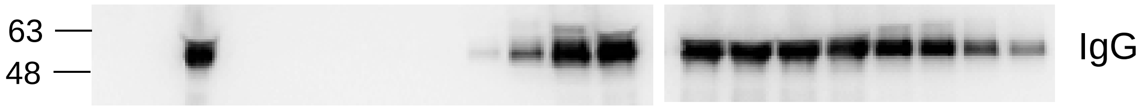

Full-length blots

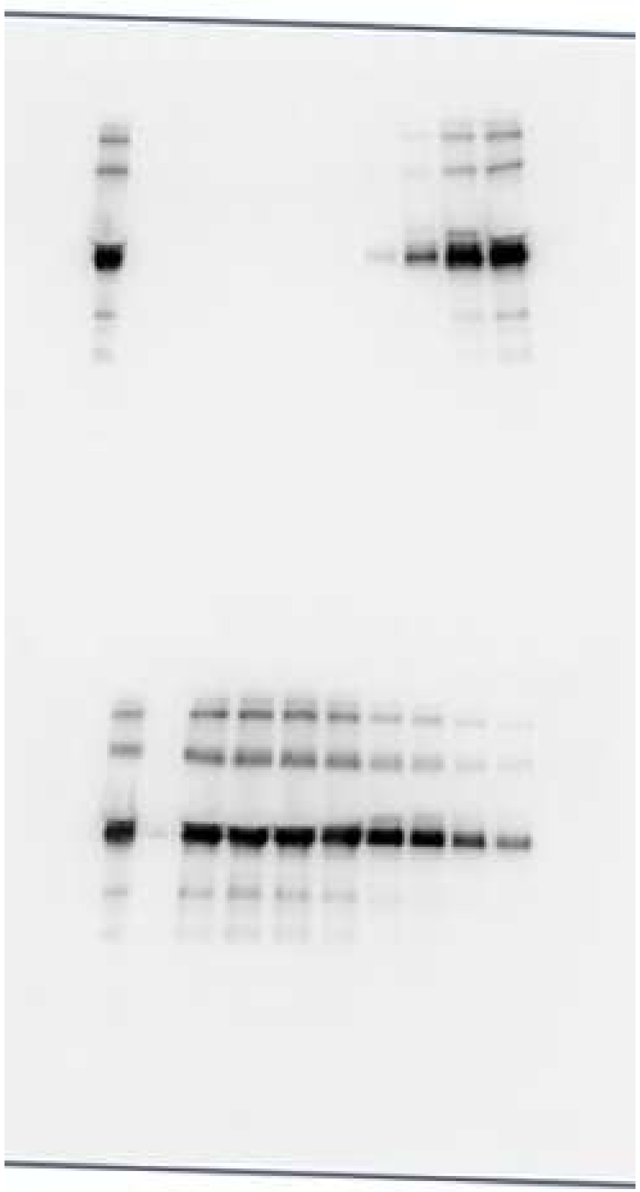

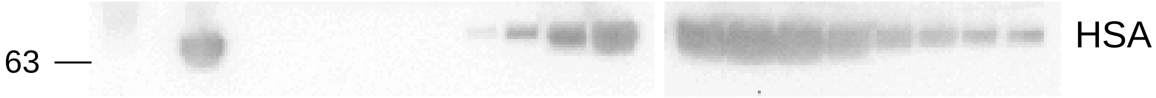

Full-length blots

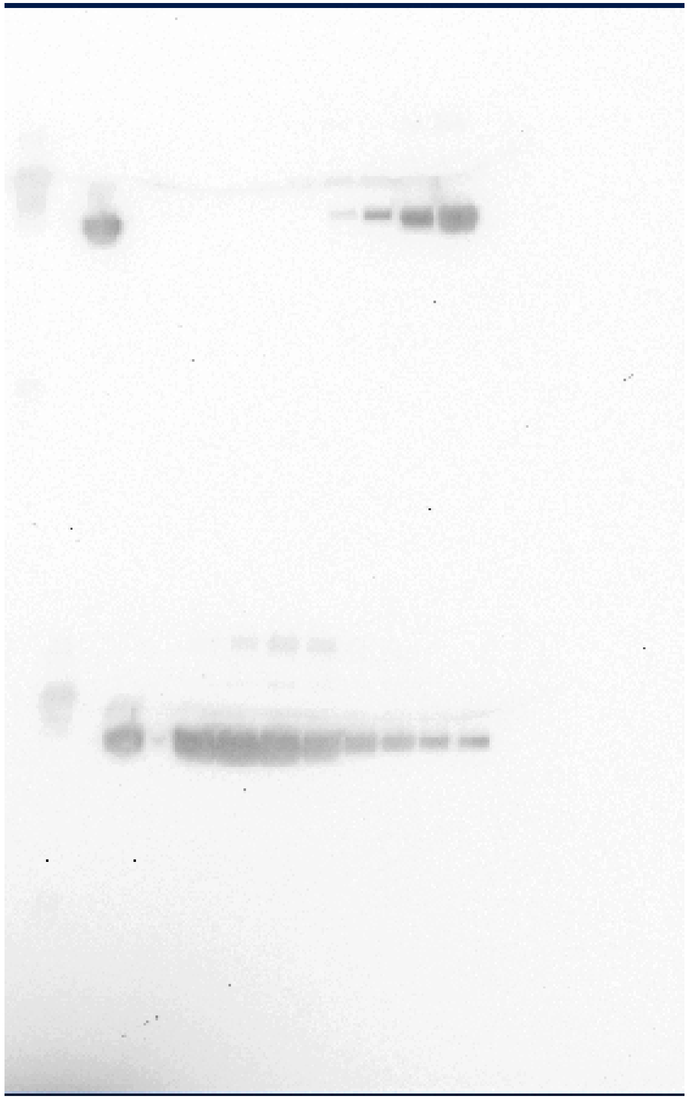

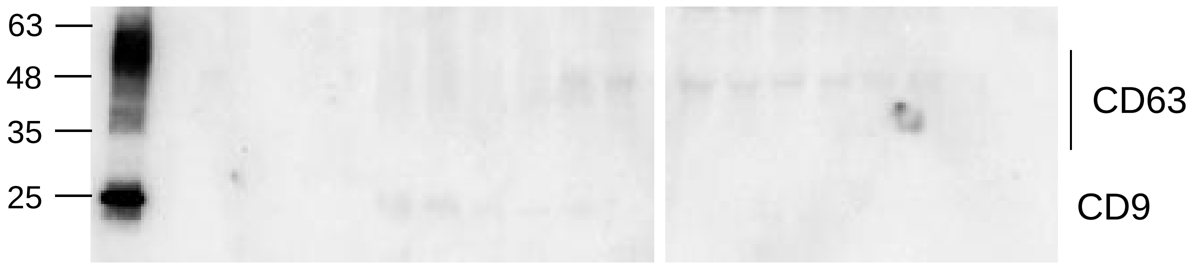

Full-length blots different exposures

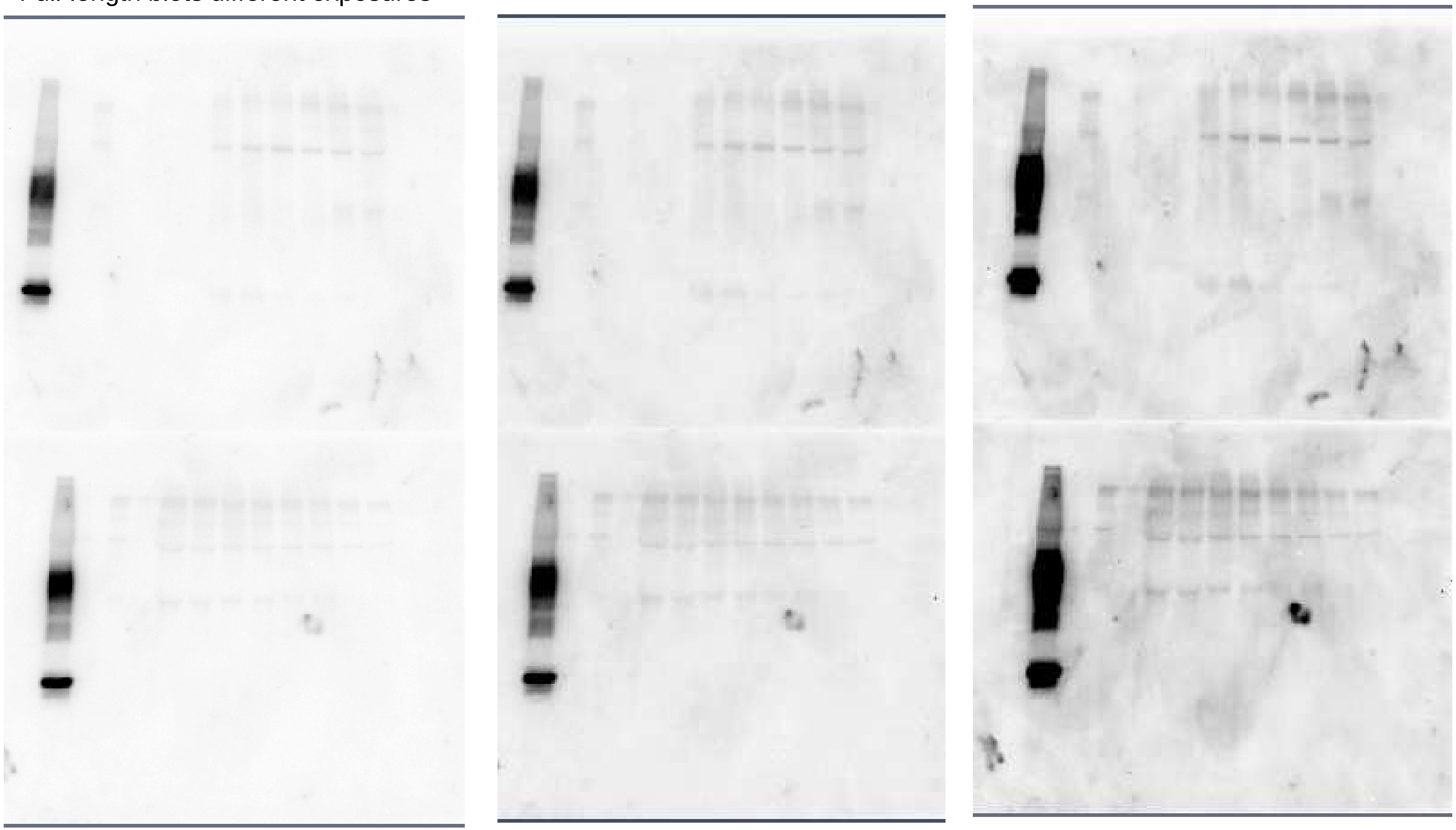

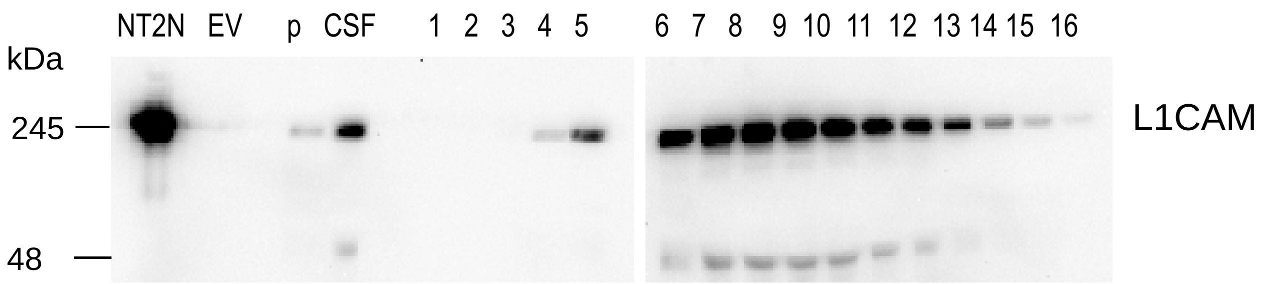

Full-length blots

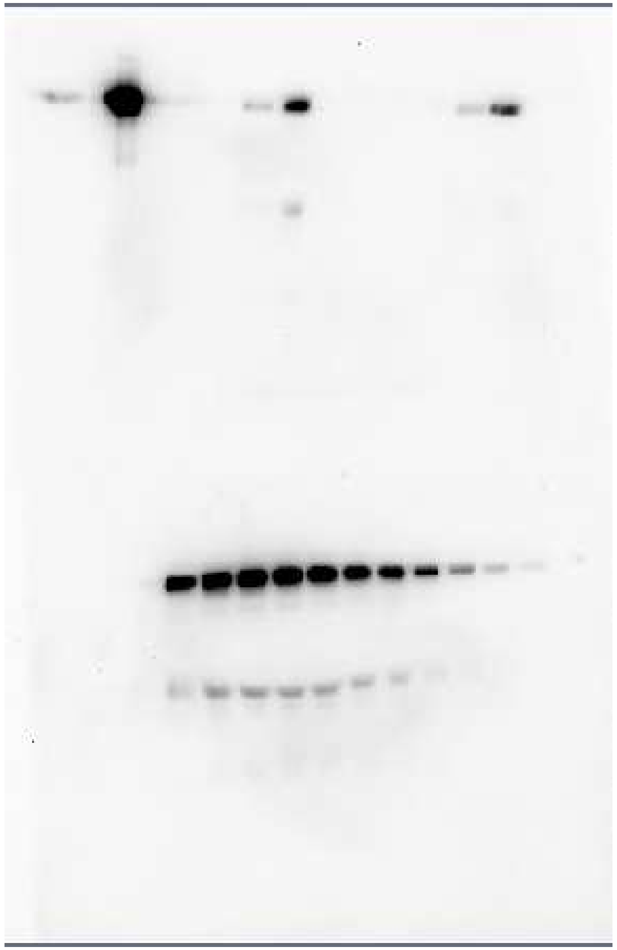

Supplement: Supplementary file 2 — Supplementaryinformation 2 [file 41598_2020_62592_MOESM2_ESM.pdf]
